# Food is medicine intervention shows promise for engaging patients attending a safety-net hospital in the Southeast United States

**DOI:** 10.3389/fpubh.2023.1251912

**Published:** 2023-10-03

**Authors:** Caroline Owens, Miranda Cook, Joy Goetz, Leslie Marshburn, Kathy Taylor, Stacie Schmidt, Jada Bussey-Jones, Rosette J. Chakkalakal

**Affiliations:** ^1^Department of Anthropology, College of Arts and Sciences, Emory University, Atlanta, GA, United States; ^2^Department of Anthropology, College of Arts and Sciences, Washington State University, Pullman, WA, United States; ^3^Open Hand Atlanta, Atlanta, GA, United States; ^4^Atlanta Community Food Bank, Atlanta, GA, United States; ^5^Grady Health System, Atlanta, GA, United States; ^6^Department of Medicine, School of Medicine, Emory University, Atlanta, GA, United States

**Keywords:** food security, nutrition, food is medicine, healthcare, chronic disease, intervention, diabetes, hypertension

## Abstract

Public health organizations, including the Academy of Nutrition and Dietetics and the American Hospital Association, recognize the importance of achieving food and nutrition security to improve health outcomes, reduce healthcare costs, and advance health equity. In response, federal, state, and private agencies are increasingly seeking to fund healthcare-based interventions to address food insecurity among patients. Simultaneously, nutrition-based interventions targeting chronic diseases have grown across the United States as part of the broader “Food is Medicine” movement. Few studies have examined the successes, challenges, and limitations of such efforts. As Food is Medicine programs continue to expand, identifying common approaches, metrics, and outcomes will be imperative for ensuring program success, replicability, and sustainability. Beginning in 2020, the Food as Medicine (FAM) program, a multipronged, collaborative intervention at Grady Health System has sought to combat food insecurity and improve patient health by leveraging community resources, expertise, and existing partnerships. Using this program as a case study, we (1) outline the collaborative development of the FAM program; (2) describe and characterize patient engagement in the initial 2 years; and (3) summarize strengths and lessons learned for future hospital-based food and nutrition programming. As this case study illustrates, the Food as Medicine program provides a novel model for building health equity through food within healthcare organizations.

## Introduction

Food insecurity, a state in which an individual or household lacks social, economic, or physical access to nutritious foods to support a healthy and active life, impacts approximately 11% of all individuals in the United States (1). Food insecurity disproportionately affects marginalized groups in the US, including those experiencing poverty (2), unstable housing (3), and among racial and ethnic minority groups (4). Studies demonstrate a negative relationship between food insecurity and diet quality in North America (5–7) including recent systematic reviews demonstrating negative associations between food insecurity and dietary patterns recommended for cardiometabolic health, including Dietary Approaches to Stop Hypertension and the Mediterranean diet, particularly among women (8). In addition, food insecurity and associated social determinants of health may compound barriers to healthcare access (3). In the face of such barriers, numerous studies have documented associations between food insecurity and adverse health outcomes, including hypertension (9–11), diabetes mellitus (12, 13), and cardiovascular disease risk (14). As a consequence of these co-morbid conditions, individuals living with food insecurity also exhibit higher healthcare utilization and costs compared to those who are food secure (15–17).

As a public health concern and a prevalent social determinant of health, food insecurity is a primary target of interventions to alleviate diet-related chronic diseases. Though intervention approaches vary widely, many apply principles of the “Food is Medicine” movement (18). “Food is Medicine” programs leverage the expertise and authority of healthcare providers to encourage participation and lifestyle change among patients (18). Within this movement, Food or Produce Prescription Programs and “Fresh Food Farmacy Programs” have emerged as on-site healthcare-based interventions that may offer access to nutrition counseling, evidence-based cooking and nutrition classes, or free or subsidized nutritious foods (19). In such programs, healthcare systems often collaborate with community partners, including nonprofit organizations, to provide resources to improve food security and diet-related health outcomes and, in the long term, reduce healthcare costs and expenditures (18). Food is Medicine programs also answer prominent calls for health-systems to actively address social determinants of health and work toward achieving health equity (20, 21).

To our knowledge, this is one of few manuscripts to detail the real-world implementation of a Food is Medicine program, and to describe the characteristics and engagement of patients who enroll. The partnerships of this program enable rigorous evaluation in real-world settings to assess program outcomes. By describing program characteristics and initial outcomes, this manuscript aims to address gaps in the current literature to enhance the sustainability, scalability, and transferability of these interventions to other hospital and clinical settings. The Food as Medicine (FAM) partnership, which began in 2020, operates as a collaboration between a large hospital system in the Southeast US and local nonprofit organizations. Akin to similar interventions across the nation, the FAM program targets patients living with or at-risk for food insecurity and hypertension or diabetes mellitus.

### Context and rationale

Over 11% of individuals living in the Southeast experience food insecurity—the highest prevalence of any region in the US (1). Across the US, prominent disparities in the experience of food insecurity among historically marginalized and minoritized communities are evident; food insecurity disproportionately affects those living in households with incomes below the poverty line and Black and Hispanic households (4). The Atlanta Metropolitan Statistical Area (MSA), defined by the US Census, is the ninth largest city in the country—with a higher percentage of non-white residents than the country overall. In a recent analysis, Shannon and colleagues found high rates of food insecurity in core urban neighborhoods in the Atlanta MSA, along with increasing challenges in suburban areas (22). As their findings suggest, there remains a critical need for food security efforts across the region, particularly those that can serve as “one-stop shops” for food and medicine.

The Grady Health System is the busiest level 1 trauma center in Georgia and has over 158,000 Emergency Department visits annually. Comprised of a hospital with 853 licensed beds and six neighborhood health centers across two counties in the Atlanta MSA, Grady serves over 2,300 patients per day. The Population Health team at Grady seeks to design, deliver and coordinate care to address the critical needs of the community in accordance with overall health status. A key priority for the Population Health team is to contribute to a coordinated system of care delivery within and outside the clinical setting the team does so by working at the nexus of three key social determinants of health: housing, transportation, and food. Previous needs assessments estimated that the prevalence of food insecurity among patients attending Grady Health System is nearly four times higher than in Atlanta overall. Based on these assessments, approximately 50% of the patient population may experience food insecurity at some point in the year; moreover, since these assessments were conducted before the COVID-19 pandemic, current patient needs are likely even greater. Recognizing this need, the aims of the Food as Medicine partnership are threefold: 1. Increase access to healthy, affordable food for patients and their families, employees, visitors, and the wider community; 2. Leverage community resources and expertise to address food insecurity and chronic disease; and 3. Improve the health and overall quality of life of patients.

### Intervention development

In 2016, Grady announced the examination and alleviation of diabetes, hypertension, and social determinants of health as community health needs priorities. At this time, the early inceptions of Food as Medicine began with a pilot Fruit and Vegetable Rx Program, discussed and evaluated at length in (23). By 2017, Grady formalized the Food as Medicine partnership by executing a Letter of Intent (LOI) with community partners outlining shared goals, a plan to address food insecurity and chronic disease, and shared fundraising targets to bring the plan to fruition. In tandem, Grady implemented Supplemental Nutrition Assistance Program (SNAP) screening and food pantry referrals at a clinic site located 1.5 miles from the pantry. From 2017 to 2019, 1,119 patients were screened for SNAP and food pantry referrals; however, a low engagement with the food pantry (10%) prompted partners to redevelop the vision for FAM. Seeking to further integrate food into the clinic space, bi-monthly food distribution was established at one clinic in 2019 and construction of the Jesse Hill Market on Grady Health System’s campus, erected at the site of a former fast food restaurant, began. Over this period, partners developed a vision for a multifaceted pronged FAM program.

### Establishing critical partnerships

Grady formed the Food as Medicine (FAM) program as a collaboration between Grady Health System, Atlanta Community Food Bank, and Open Hand Atlanta. Constructing a shared vision and goals across partners proved critical to the program’s success in its inception and initial stages. A Feeding America affiliate, the Atlanta Community Food Bank has a long history of working to provide access to nutritious meals for those in need within their 29-county service area. Open Hand Atlanta, a non-profit agency, seeks to eliminate disability and untimely death due to nutrition-sensitive chronic disease and medically-tailored meals and nutrition education. By working in coordination with the Atlanta Community Food Bank and Open Hand Atlanta, the FAM program leveraged the experience and resources of existing community-based food and nutrition security resources in the development, design, and implementation of programming. Finally, partnerships with Emory University researchers enable evaluation of this program. These non-governmental, private, and research sector partnerships have proven vital to the implementation and maintenance of the FAM program over its initial 3 years. This case-study focuses on one of the integral prongs of the FAM program: The Food Prescription Program. Given the increase in food prescription programs across the U.S., Grady prioritized disseminating evaluation findings from the Food Prescription Program prong, with other aspects of the program to be evaluated and shared more widely in the future.

### Programmatic elements

The Food Prescription Program, a service line within the larger Grady FAM program, is a multi-pronged intervention that provides eligible patients with nutrition counseling, cooking classes, and fresh food (purchased from the Atlanta Community Food Bank). The Food Prescription Program operates within the Jesse Hill market space to serve as a hub for nutrition and well-being for Grady patients, employees, and the greater community. The Jesse Hill market also houses a teaching kitchen that focuses on plant-based cooking and nutrition and chronic disease education.

### Theoretical framework

**Figure 1:**
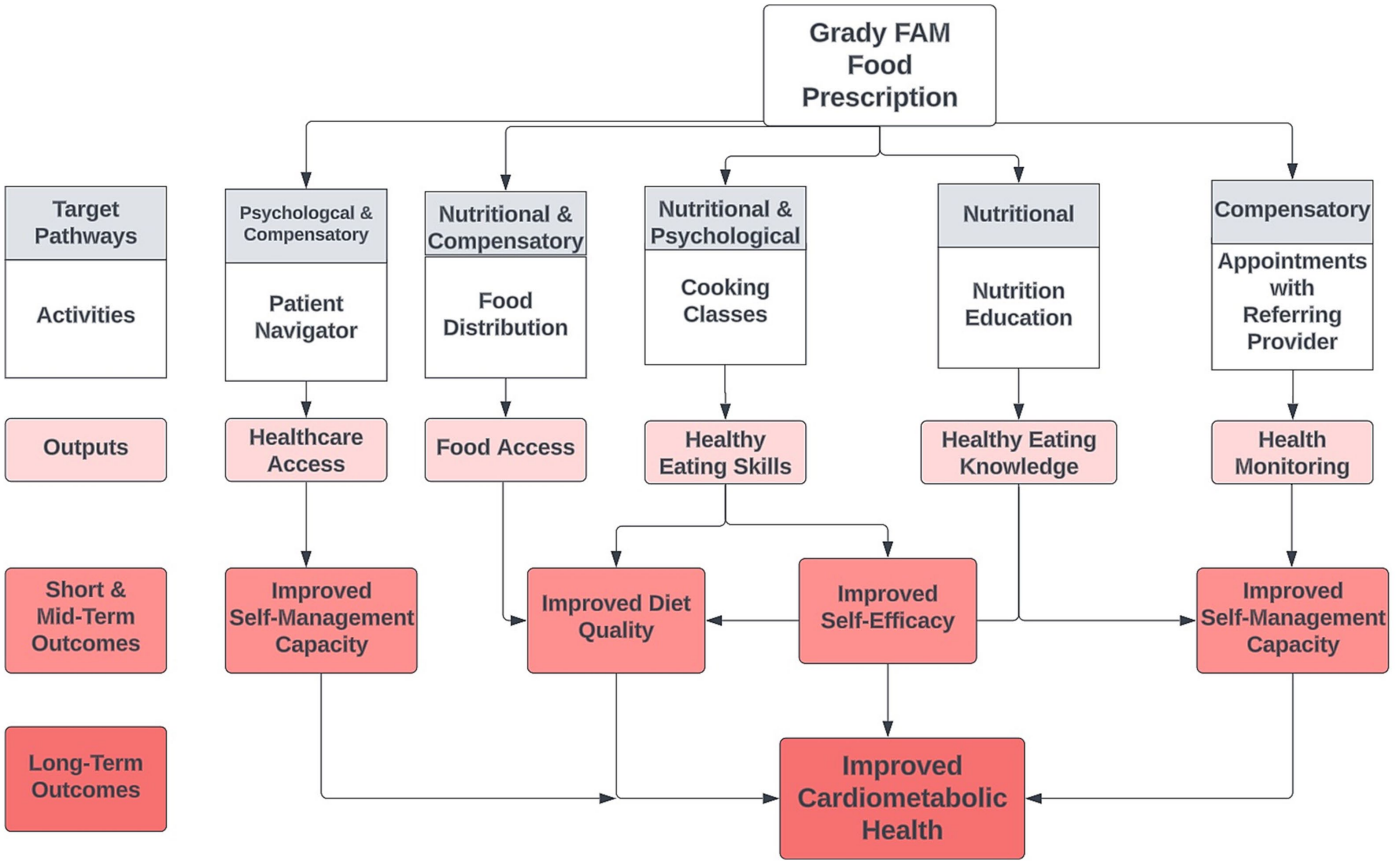
Grady food prescription program conceptual model.

Food security is often conceptualized as being comprised of four pillars: availability, accessibility, utilization, and stability. Scholarly examination of these pillars is varied, with an arguable over-representation of dimensions of availability and access in commonly used measurement tools and studies of insecurity and health. The Food Prescription Program attempts to address multiple pathways proposed to underlie food insecurity and adverse health outcomes, including nutritional, compensatory, and psychological pathways, as outlined by Te Vazquez and colleagues in a recent systematic review (24). The nutritional pathway connecting food insecurity with chronic disease occurs through constrained dietary options and lower diet quality, specifically lower consumption of fruits, vegetables, whole grains, and lean meats (5–7, 13, 25). Alongside nutritional and dietary constraints, compensatory measures such as trade-offs between food and medications or other basic needs may reduce capacities to manage well-being or existing conditions (26). Psychological factors, including depression, anxiety, and feelings of shame or embarrassment, may increase experiences of psychosocial and physiological stress and decrease self-efficacy (27–31). Many scholars suggest that these pathways collectively promote cycles of insecurity and disease. As shown in Figure 1, the Grady Food Prescription Program attempts to address these mechanisms through key program outputs: food distribution, cooking classes, nutrition counseling with a dietitian, and appointments with a primary care provider. Receipt of fresh fruits and vegetables, whole grains, and plant-based proteins meats intends to simultaneously reduce dietary constraints and improve diet quality, which dietitians and instructors bolster through nutrition and food preparation knowledge. The knowledge and education components of the program also work to improve patients’ self-efficacy, targeting the psychological pathway. Finally, appointments with the provider enable more monitoring of health and pre-existing conditions to improve capacities for self-management. The Food Prescription Program aims to simultaneously improve food security and cardiometabolic health through these outputs and program outcomes.

## Methods

### Recruitment

Eligibility for the program is determined by healthcare providers during routine outpatient clinic visits. Patients are eligible for a Food Prescription Program prescription if they: (1) screen positive for food insecurity using the validated two-item Hunger Vital Sign™ (HVS) and (2) are identified as having uncontrolled hypertension (systolic blood pressure greater than 140 mmHg *or* diastolic blood pressure greater than 90 mmHg during the last measure taken) or uncontrolled diabetes Type 1 or 2 (last hemoglobin A1c reading greater than 9.0). Notably, the HVS™ identifies risk for food insecurity; therefore, individuals who are living with marginal food security may screen as “at-risk” for food insecurity using the HVS™. Studies in clinical settings show that the HVS shows high sensitivity and specificity with the Household Food Security Survey Module (32, 33), which the Food Prescription Program staff uses to assess food insecurity. Grady Health System began screening for Social Determinants of Health (SDOH), including food insecurity, in 2019. The SDOH screening occurred in a phased process, such that 11 primary care and diabetes clinics conducted screening between August 2020 and August 2022; however, Grady has since expanded SDOH screening to all outpatient clinics as of June 2023.

To simplify referral, the team developed a Best Practice Alert (BPA) within Epic (electronic medical record system) which prompts providers to sign a referral (i.e., prescription) to the Food Prescription Program. As a feature of the electronic medical record, referral to the Food Prescription Program is a staff-led intervention, offering nurses and certified medical assistants the ability to respond to the BPA. In the period between August 2020 and August 2022, each month an average of 236 BPAs were prompted, 122 referrals were made, and 42 enrollments occurred. Once enrolled, food prescriptions provide patients access to the Food Prescription Program for a 3-month “episode” of care which can be renewed up to 3 times (i.e., 1 year of access to the Food Prescription Program). During each episode, patients are invited to pick up fresh produce boxes biweekly, attend cooking classes in Jesse Hill Market’s on-site Teaching Kitchen, participate in nutrition education sessions (one-on-one, group, or telehealth) with a registered dietitian, and continue to follow-up with their primary healthcare provider. The food prescription process is documented within the electronic medical record (EMR) system, including the referral and enrollment for eligible and participating patients, documentation of visits to the Food Prescription Program, Teaching Kitchen and nutrition education sessions, and health records. At their first Food Prescription Program visit, patients complete a standardized survey that further assesses social determinants of health using validated instruments. Specifically, patients complete the 6-item Household Food Security Survey Module (HFSSM), the Center for Disease Control Healthy Days Core Module (34), a one-item screening for housing insecurity, and information on household composition and utilization of food assistance. In addition to data from the survey instrument, a qualitative phone-based survey was conducted in April 2022 among a subset of participants to query barriers to program engagement and retention. To explore how these sociodemographic and health characteristics vary by loss to follow-up and re-enrollment, we divide descriptive tables into columns, as follows: the first column displays data on patients who did not renew the prescription after their first 3-month episode (those lost to follow-up), the second column displays data on those who renewed their prescription, the third column displays data from all patients who enrolled in the Food Prescription Program and were eligible for renewal, and the final column displays test statistics and *p*-values for statistical tests comparing patients who did not renew their prescription to those who renewed their prescription. Chi-square tests were used to assess differences across categorical variables and independent samples t-tests were used for continuous variables. We use an alpha level of 0.05 to determine statistical significance. To our knowledge, this is one of few Food is Medicine programs to be fully integrated as a clinical service line within the EMR in a US healthcare system.

## Results

### Patient characteristics and engagement

Between August 2020 and August 2022, 1,012 patients visited the Food Prescription Program at least once. Of those, 863 were eligible to renew their prescription by August 2022 based on their initial start date. During this period, the Food Prescription Program distributed over 142,000 pounds of food to patients enrolled in FAM. Overall, approximately 42.6% of patients renewed their prescription. Reflecting the demographics of the hospital, the majority of enrolled these patients identify as Black or African American (93%) and female (60%). Notably, 20% of these patients experienced housing insecurity in the previous 12 months, illustrating the influence of multiple social determinants on the health of this community. Similarly, using the Household Food Security Survey Module, nearly 70% of all enrollees experienced low or very low food security in the past month. Enrollees ranged in age from 19 to 90 years, with an average age of 56 years. Approximately one-third of all enrollees reported living in a household with at least one child, while almost half reported living in a household with at least one adult over age 60. Most enrollees reported preparing their own meals at home, which indicates capacity to implement lessons from the cooking and nutrition education components of the Food Prescription Program.

As shown in Table 1 significantly greater proportion of participants who renewed the initial prescription were female and had older adults living in their household compared to participants who did not renew the initial prescription. As shown in Table 1, significantly more men did not renew the initial prescription compared to those who did renew the initial prescription. Additionally, mean age was significantly lower among those who did not renew the initial prescription compared to patients who renewed. Similarly, those with at least one older adult in the household were significantly more likely to renew their prescription compared to those without older adults in the household. However, there were no other significant demographic or household composition differences observed between those who renewed their prescription completed the program and those who were lost to follow-up.

**Table 1:** Sociodemographic characteristics at baseline.

	Initial prescription not renewed (*N* = 495)	Initial prescription renewed (*N* = 368)	Overall eligible (*N* = 863)	Test statistic, *P*
Gender				8.87, 0.003
Female	276 (55.8%)	243 (66.0%)	519 (60.1%)	
Male	219 (44.2%)	125 (34.0%)	344 (39.9%)	
Age (in years)				−4.12, <0.001
Mean (SD)	54 (± 11)	57 (± 9.9)	56 (± 11)	
Race				7.01, 0.072
Black or African American	456 (92.1%)	348 (94.6%)	804 (93.2%)	
Hispanic	14 (2.8%)	4 (1.1%)	18 (2.1%)	
Multi-Racial, Other, or Unknown	8 (1.6%)	10 (2.7%)	18 (2.1%)	
White	17 (3.4%)	6 (1.6%)	23 (2.7%)	
Ethnicity				0.052
Hispanic	18 (3.6%)	6 (1.6%)	24 (2.8%)	
Non-Hispanic	475 (96.0%)	356 (96.7%)	831 (96.3%)	
Unknown	2 (0.4%)	4 (1.1%)	6 (0.7%)	
Patient refused	0 (0%)	2 (0.5%)	2 (0.2%)	
Household demographics				
Any children (yes)	168 (33.9%)	107 (29.1%)	275 (31.9%)	2.56, 0.11
Missing	37 (7.5%)	23 (6.3%)	60 (7.0%)	
Any older adults (yes)	217 (43.8%)	191 (51.9%)	408 (47.3%)	4.10, 0.043
Missing	29 (5.9%)	14 (3.8%)	43 (5.0%)	
Food insecurity status				1.63, 0.44
High or marginal food security	117 (23.6%)	92 (25.0%)	209 (24.2%)	
Low food security	165 (33.3%)	110 (29.9%)	275 (31.9%)	
Very low food security	172 (34.7%)	141 (38.3%)	313 (36.3%)	
Missing	41 (8.3%)	25 (6.8%)	66 (7.6%)	
Was there a time in the last 12 months when you did not have your own place to stay were homeless or stayed in a shelter?				2.61, 0.11
Yes	112 (22.6%)	66 (17.9%)	178 (20.6%)	
Missing	18 (3.6%)	13 (3.5%)	31 (3.6%)	
When you eat at home who usually prepares meals?				2.59, 0.11
Other	71 (14.3%)	39 (10.6%)	110 (12.7%)	
Self	391 (79.0%)	309 (84.0%)	700 (81.1%)	
Missing	33 (6.7%)	20 (5.4%)	53 (6.1%)	

Table 2 displays baseline biometrics of those who did not renew their prescription, those who renewed their prescription, and all eligible enrollees. Of those who enrolled in the food prescription, 88% had elevated or hypertensive blood pressure (systolic blood pressure greater than 140 mmHg *or* diastolic blood pressure greater than 90 mmHg), and 48% had a baseline HbA1c greater than or equal to 9.0%. Approximately 58% of patients had hypertensive blood pressure readings *and* HbA1c values of greater than or equal to nine, suggestive of a high prevalence of comorbidity relative to the general population. Those lost to follow-up had significantly smaller baseline waist circumference, but significantly greater diastolic blood pressure and A1C levels compared to than those who renewed prescriptions. There were no other differences in baseline physical or perceived health measures between the groups, suggesting that program retention may not be affected by differences in morbidity status or baseline health condition. As shown in Table 3, most enrollees (48%) rated their perceived health as “fair,” with less than 10% of enrollees rating their health as “excellent” or “very good” (2 and 5%, respectively) at baseline. Using the CDC Healthy Days Tools, enrollees reported 11 days of poor physical health, 8 days of poor mental health, and 8 days during which mental or physical health prevented usual activities during the last month, on average.

**Table 2:** Biomarker health characteristics at baseline.

	Initial prescription not renewed (*N* = 495)	Initial prescription renewed (*N* = 368)	Overall eligible (*N* = 863)	Test statistic, *P*
Waist circumference (ins)				−2.12, 0.035
Mean (SD)	41 (± 8.1)	42 (± 8.0)	41 (± 8.1)	
Missing	122 (24.6%)	79 (21.5%)	201 (23.3%)	
Body mass index (kgm^2^)				−1.36, 0.17
Mean (SD)	32 (± 8.6)	33 (± 8.7)	33 (± 8.7)	
Missing	7 (1.4%)	8 (2.2%)	15 (1.7%)	
Systolic blood pressure (mmHg)^1^				0.11, 0.92
Mean (SD)	140 (± 20)	140 (± 20)	140 (± 20)	
Missing	5 (1.0%)	11 (3.0%)	16 (1.9%)	
Diastolic blood pressure (mmHg)				3.04, 0.002
Mean (SD)	82 (± 12)	79 (± 12)	81 (± 12)	
Missing	5 (1.0%)	11 (3.0%)	16 (1.9%)	
A1C (%)				2.57, 0.010
Mean (SD)	9.4 (± 3.2)	8.9 (± 2.9)	9.2 (± 3.1)	
Missing	16 (3.2%)	17 (4.6%)	33 (3.8%)	

^1^Individuals with systolic blood pressure greater than 140 mmHg or diastolic blood pressure greater than 90 mmHg were classified as having hypertension. Individuals with A1C greater than or equal to 9.0% were classified as having uncontrolled diabetes.

**Table 3:** Perceived health characteristics at baseline.

	Initial prescription not renewed (*N* = 495)	Initial prescription renewed (*N* = 368)	Overall eligible (*N* = 863)	Test statistic, *P*
In general, how would you describe your health?				1.66, 0.80
Excellent	11 (2.2%)	7 (1.9%)	18 (2.1%)	
Very good	23 (4.6%)	24 (6.5%)	47 (5.4%)	
Good	121 (24.4%)	91 (24.7%)	212 (24.6%)	
Fair	240 (48.5%)	171 (46.5%)	411 (47.6%)	
Poor	89 (18.0%)	65 (17.7%)	154 (17.8%)	
Missing	11 (2.2%)	10 (2.7%)	21 (2.4%)	
How many days did poor mental or physical health prevent you from doing your usual activities?				−1.76, 0.08
Mean (SD)	7.8 (± 11)	9.2 (± 11)	8.4 (± 11)	
Missing	27 (5.5%)	18 (4.9%)	45 (5.2%)	
How many days during the last 30 days was your physical health poor?				−0.61, 0.54
Mean (SD)	11 (± 12)	11 (± 12)	11 (± 12)	
Missing	30 (6.1%)	19 (5.2%)	49 (5.7%)	
How many days during the last 30 days was your mental health poor?				−0.38, 0.70
Mean (SD)	8.4 (± 11)	9.2 (± 11)	8.5 (± 11)	
Missing	31 (6.1%)	18 (4.9%)	50 (5.8%)	

### Barriers to engagement

Of the 863 individuals who attended the Food Prescription Program at least once were eligible to renew their prescription, 495 (57.4%) did not renew their prescription to re-enroll in the Food Prescription Program. Noting that many participants enrolled had not met the participation requirements to re-enroll in an additional episode, a qualitative phone-based survey was conducted to query barriers to program engagement in April 2022. We identified 62 participants who would have been eligible for re-enrollment at this point had they met requirements and were able survey 25 (40% response rate). Barriers to engagement identified were highly individual but were often related to competing priorities and overlapping social determinants of health such as lack of transportation, caregiver responsibilities, work hours, and physical health challenges. Some responses were not able to be categorized but generally discussed the time commitment involved in meeting program requirements within the 3-month episode window (24% of responses discussed the time commitment as a barrier) (Table 4).

**Table 4:** Summarized barriers to engagement.

Theme	Frequency n (%)	Illustrative quote
Transportation issues	11 (44%)	“Just availability at the times they wanted to do certain things, like the times they wanted to do the cooking classes, I did not have a way to get there.”
Caregiver responsibilities	4 (16%)	“I have a disabled daughter and she has been having complications and it was hard for me to participate and take her back and forth from the clinic.”
Work hours	1 (4%)	“Because of the simple fact that I had to work.”
Physical health challenges	3 (12%)	“I was having [2 chronic health conditions] … I’m talking fatigue that hits you like no other. I’ll be shopping and feel like I’m ‘bout to pass out. I’m trying to learn everything I can though.”

## Discussion

Following principles of the Food is Medicine movement, this study of the Grady Food as Medicine program development and delivery is shared here for the purposes of transparency, replicability and transferability, and the enhancement of public health impact by integrating resources to alleviate social determinants of health directly within a health system. During the first 2 years of a Food Prescription program, Grady Health System engaged 1,012 patients living with diabetes or hypertension and at-risk for food insecurity, retaining approximately 42.6% of those eligible for future iterations of the program. The significantly greater loss to follow-up among individuals who identified as male warrants further investigation. In a recent study, Sauder and colleagues report similar findings from the Diabetes Prevention Program, in which older men and younger men were significantly less likely to complete one or more sessions than older women and younger women (35). Analyses of trends in home cooking demonstrate that a greater proportion of females report cooking at home. Furthermore, while the percentage of males who report cooking at home has increased overall in recent years, changes vary by educational attainment. Specifically, Taillie reports that the percentage of males with less than a high school education who cook has remained stagnant over the past decade (36). It is possible that documented gender norms surrounding cooking and feeding responsibilities explain the greater loss to follow-up among men, though gendered themes did not emerge from our qualitative investigation. In this vein, sociologists, including Fielding-Singh and Oleschuk propose that nutrition disparities between the sexes may, in part, derive from these gendered norms of “foodwork”—the practices that support and facilitate eating within households (37). As has elsewhere been argued, these structural and societal dimensions must be attended to in the design, implementation, and evaluation of nutrition interventions, including Food is Medicine programs (38). Akin to our findings, qualitative research on similar programs suggests that economic and structural barriers, such as limited income, caregiver responsibilities, and medical concerns associated with disease management may hinder program engagement. In addition to these key findings, program strengths, lessons learned, and additional recommendations for other healthcare systems are highlighted below.

### Strengths

The intervention development process strived to build upon effective research-community partnerships, incorporating community organizations working in the local food system sector for several decades and leveraging research expertise through academic partnerships with local universities. These organizations afforded the FAM partnership access to valuable networks and funding, without which this program would not be possible. The BPA alerts within the electronic medical record and nurse-led protocols facilitate easy identification of eligible participants, timely referral, and clinical integration of this program within the health system. Integrating referral in the electronic medical record also enables data sharing across clinical and intervention spaces. Moreover, the Food as Medicine program attempts to address the four major pillars of food insecurity: availability, access, stability, and utilization. While concerted efforts to address issues of availability and access are evident in intervention approaches, the cooking classes work to also improve utilization and patients’ ability to re-enroll for up to 1 year aims to improve stability. Furthermore, the Food as Medicine program acts as a vital “one-stop-shop,” for healthcare and food. The physical infrastructure and proximity of the Food Prescription Program in relation to the hospital alleviates some barriers to enrollment. FAM also enables patients to visit the Food Prescription Program on the same day as their initial referral, enabling patients to access food immediately. Nevertheless, as is evident in the proportion of people who do not return for a second visit, there remains space for growth regarding engagement and retention.

### Lessons learned and future directions

In the future, Grady aims to further develop the Food Prescription Program and the overarching FAM program, with a particular emphasis on improving referrals and alleviating barriers to program participation. Based on our preliminary evaluation, engagement with the program appears to be representative of the patient population at Grady, though a fraction of those eligible enroll in the FAM program. Further research is needed to understand barriers to engagement across each level of programmatic implementation. In this regard, more work should focus on barriers within the referral system, including variability across healthcare provider and clinical referral practices. Similarly, challenges or resistance to program enrollment among those referred warrants further attention. Among those who enroll in FAM, re-enrollment for additional three-month increments remains low. This begs the critical question: Why do people not remain engaged? And What additional supports can health systems implement to reduce barriers to engagement? Preliminary findings from brief interviews with enrollees suggest that transportation assistance may improve engagement and retention. Critically, program design anticipated that lack of access to transportation would pose a barrier for patients, particularly for bi-weekly food pick-ups. To address this barrier, Grady has piloted two transportation support programs in conjunction with the FAM program: home delivered boxes directly from the Atlanta Community Food Bank and car share rides for FAM participants funded by a health plan partner. In that regard, ongoing research with academic partners seeks to understand the added value of transportation assistance and incentives.

Other healthcare systems interested in developing Food is Medicine partnerships should prioritize the early establishment of cross-sector partnerships spanning nongovernmental and academic organizations with vested interest in the community. Additionally, programs should consider funding sources and funding sustainability. Program costs will vary depending on established partnerships and target patient engagement; at present, funding for Food is Medicine programs may incorporate governmental and private funds. As noted in our limitations section, patient engagement may present unique and contextually dependent challenges to program success. The pre-existence of social determinants of health screening facilitated recruitment for FAM at Grady and may provide a useful scaffold for enrollment in food and nutrition security programming within other healthcare systems. Finally, health systems and advocates alike must work toward a paradigm shift in how food and nutrition are treated and covered. More specifically, by viewing healthy foods, including fruits and vegetables, as fundamental to well-being and preventative care, health systems may promote increased coverage of these programs by insurance payors.

### Global perspectives

Though Food is Medicine programs are most prominent in North America, the lessons learned have global relevance. A Food is Medicine program offers insight into how a more holistic approach to food and eating can sustainably improve well-being. The program emphasizes dietary quality in addition to quantity in a manner that seeks to address each of the pillars of food insecurity, including those often unaddressed in other programs, such as utilization and stability. One of the future directions of this program—increasing emphasis on culturally preferred and culturally relevant foods derives from the premise that celebrating foodways is essential for combatting the often-racialized stigmatization of certain foodways and for generating more sustainable dietary change. From the standpoint of sustainability and resilience, it is also important to reference and incorporate produce and cultural foods grown locally, sustainably. Relatedly, programs that adopt more multifaceted approaches, including access to community gardens and arable land, which can foster physical activity and social connection—both shown to reduce rates of mood disorders (39, 40)—can be replicated across many nutrition interventions in many global contexts. With growing concern over the impact of climate change on global food security, the future of resilient communities may depend on these integrated and more localized approaches.

## Conclusion/broader impacts

In response to the disproportionate burden of food insecurity affecting the patient population at a large safety-net health system in the Atlanta MSA, Grady collaboratively developed a healthcare-integrated Food as Medicine program to improve food access and patient well-being. This case study details the development, refinement, and initial findings regarding patient engagement. In so doing, it aims to facilitate the replication or transferability of Food as Medicine interventions toward improving food security and human well-being for patients nationwide.

## Data availability statement

The datasets presented in this article are not readily available because they include public health information. Requests to access the datasets should be directed to lhmarshburn@gmh.edu.

## Ethics statement

The studies involving humans were approved by the Emory University Institutional Review Board; Grady Health Research Oversight Committee. The studies were conducted in accordance with the local legislation and institutional requirements. The ethics committee/institutional review board waived the requirement of written informed consent for participation from the participants or the participants’ legal guardians/next of kin because this study was deemed quality improvement and program evaluation.

## Author contributions

CO: conceptualization, writing-original draft, and analysis. MC: analysis. JG: implementation. LM and KT: project administration. SS and JB-J: resources and supervision. RC: conceptualization, resources, and supervision. All authors contributed to review and editing of the manuscript.
